# Seizing opportunities and navigating the challenges of spaceflight research as an early career researcher

**DOI:** 10.1113/EP091469

**Published:** 2025-05-05

**Authors:** Colleen S. Deane

**Affiliations:** ^1^ Human Development & Health, Faculty of Medicine University of Southampton, Southampton General Hospital Southampton UK

## 1 INTRODUCTION

Humankind has entered a new chapter of deep space exploration, with private companies and government agencies internationally vying to sustainably inhabit the Moon and Mars, providing more flight opportunities than ever before. Critical to the success of these long‐term spaceflight missions is the core scientific team, particularly the involvement of early career researchers (ECRs) who often perform the experiments, ensuring the scientific objectives are met. While the spaceflight field represents a unique and alluring research environment for ECRs, it is also a challenging environment to establish and maintain a productive research career. This editorial explores the key challenges facing ECRs wanting to engage in spaceflight research and the many opportunities that ECRs can capitalise on to establish themselves as future leaders in the field. For the purposes of this editorial, an ECR is defined as someone within 8–10 years of receiving their PhD.

## 2 CHALLENGES

### 2.1 How can ECRs get into the field in the first place?

Often the biggest hurdle facing ECRs is breaking into the spaceflight field in the first place, particularly in the absence of any pre‐existing links with spaceflight researchers. This is where being proactive to establish strong multi‐disciplinary networks is essential. This can be achieved in many ways. First, attending flagship conferences, such as the International Astronautical Congress (IAC) and Space‐Comm Expo, is a great opportunity for ECRs to meet with spaceflight research leaders, mentors and industry leaders. Second, becoming a member of internationally recognised societies, such as the American Astronautical Society, will similarly enable access to key events and mentors. Third, ECRs with expertise in a different field but looking to bridge with spaceflight (e.g., a bioinformatician wanting to apply their skillset to space‐flown samples) can participate in ESA‐ and NASA‐affiliated working groups (e.g., NASA Open Science Data Repository (OSDR) Analysis Working Groups), where opportunities exist to be involved in projects, writing papers and attending meetings. For those ECRs that already have pre‐existing spaceflight connections, it is important to get as much exposure to real spaceflight experiments and/or datasets as possible.

### 2.2 If ECRs can access the field, how do they fund it?

ECRs with established multi‐disciplinary networks will look to secure funding to support their spaceflight experiment and analysis. This step is possibly the most gruelling. First of all, ECRs must be aware that the funding streams to support spaceflight research vary between countries and can be challenging to navigate (for a comprehensive overview of European funding schemes, see Deane et al., 2022). For example, traditionally in the UK, funding to support space biology experiments requires a double peer review system, whereby researchers must first apply via ESA for access to space, and if successful, a second application to the UK Research Councils (UKRI) is required to fund the science exploitation. The second step is complex since space biology falls outside of UKRI's remit; thus, the science case must centre on translational Earth benefits to be successful (e.g., spaceflight as an analogue in which to study human ageing on Earth). With the rapid privatisation of space and increased governmental interest (e.g., from UKSA), the funding landscape is continually and rapidly changing, requiring ECRs to stay up to date. Additionally, the grant process itself is often too complex, especially for ECRs in isolation with no prior experience. As an example, the grant process may involve unfamiliar processes such as market breakdowns, subsidy controls and advanced governance, thus requiring comprehensive expert guidance from senior researchers with experience in these applications. Due to the competitive nature of these grants, a critical consideration is whether ECRs are competitive enough to lead these applications or whether they need to be led by a senior spaceflight researcher with an already proven track record. To overcome this, ECR‐specific funding calls would help champion emerging leaders in the field and support the vision of long‐duration missions, however, such calls do not yet exist.

### 2.3 If ECRs can fund their spaceflight research, how do they make it a sustainable and successful career?

The historically slow cadence of spaceflight launches has meant that leading spaceflight researchers have a spaceflight experiment roughly every 5–10 years. This is a major bottleneck for ECRs on fixed‐term contracts, since a single fixed‐term contract is unlikely to allow the ECR to see the project from conception through to launch. This is exacerbated further by mission delays – which are common in spaceflight due to technical or budgetary setbacks – and can jeopardise project outputs (e.g., publications), data generation for follow‐on hypothesis‐driven project proposals and career milestones (e.g., permanent academic position). To mitigate this, ECRs must diversify their research portfolio, balancing microgravity‐based projects with parabolic and/or ground‐based studies that may yield faster outputs. Successful spaceflight researchers often also have an Earth‐based research theme that complements their spaceflight interests and acts as a vehicle for bi‐directional translation (e.g., research into ageing).

## 3 OPPORTUNITIES

### 3.1 Spaceflight networks are growing

With the exponential interest in making interplanetary habitation a reality, there are growing numbers of networks and working groups bringing together scientists of mixed career stages and scientific backgrounds to rapidly expedite and exploit spaceflight science. To highlight a few, there are the ESA Topical Teams, which are groups of scientists formed around selected topics; there are the ESA Working Groups, which are groups of science experts who report to ESA advisory committees on matters concerning their specific area; and there are the already mentioned NASA OSDR Analysis Working Groups, which are collections of interested parties who provide feedback on scientific standards, establish analytical processes and publish insights using OSDR data. These groups (and there are many more) have produced several co‐developed publications (e.g., Deane et al., 2022; Manzano et al., 2023; Overbey et al., 2024) and grant applications and thus represent fantastic thought networks for ECRs to join.

### 3.2 Spaceflight opportunities are increasing

To support the bold ambitions of going back to the Moon and onward to Mars (e.g., ESA Strategy 2040), industry and government have invested record levels of funding into the spaceflight sector. This is reflected in the variety of financial support now available, including regular announcements of opportunities from ESA and UKSA and the NASA funded Translational Research Institute for Space Health (TRISH) and the Human Research Programme (HRP). Recently, there have also been purely commercial funding opportunities (e.g., Space X and Vast joint call). Thus, while the funding landscape is complex and challenging to navigate, the number of opportunities available to ECRs is better than ever.

### 3.3 Spaceflight research for benefits on Earth

While spaceflight research can be challenging and slow to see fruition, the impact on technology and health on Earth can be seismic. To provide a couple of examples, (i) imaging technology used in the Hubble Space Telescope to detect distant stars was adapted for the detection of breast cancer (digital mammography), and (ii) coating originally designed for astronaut helmet visors to protect against harmful UV rays and glare was adapted for sunglasses on Earth. This translational element of spaceflight research can significantly benefit ECRs' research careers and opportunities via the creation of spin‐off companies, industry partnership prospects, diverse research funding opportunities and policy influence.

### 3.4 Spaceflight inspires the next generation

The inherently fascinating nature of spaceflight exploration has the power to captivate and engage the younger generations. Recognising the importance of engaging with and inspiring the next generation, ESA named ‘Inspire Europe’ as one of its five goals of their ESA Strategy 2040 (European Space Agency, 2025). This represents a fantastic opportunity for ECRs to maximise their spaceflight experiments/experience via engaging in outreach. To develop an outreach and engagement profile, ECRs can take part in podcasts, media interviews, write lay magazine articles, record docuseries, create interactive activities for outreach events and create classroom activities.

## 4 CONCLUSION

The spaceflight field has never been more active and vibrant, providing more opportunities than ever for ECRs to get involved in spaceflight research. Key challenges facing ECRs include funding and access to spaceflight platforms, which should and could be remedied by the rapid growth and privatisation of the spaceflight sector. However, significant progress in harmonising the funding streams and streamlining the application process is needed to encourage ECRs to successfully engage with the process. Principal investigators of long‐term spaceflight missions are encouraged to prioritise the training of ECRs in all relevant avenues of spaceflight research (e.g., conducting experiments in the microgravity setting, grant capture) to help bring through the next generation of thought leaders in the field, ensuring the continued success of interplanetary space research.

## AUTHOR CONTRIBUTIONS

Sole author.

## CONFLICT OF INTEREST

None declared.

## FUNDING INFORMATION

No funding was received for this work.
